# Faecal sludge pyrolysis as a circular economic approach to waste management and nutrient recovery

**DOI:** 10.1007/s10668-023-04219-4

**Published:** 2023-11-30

**Authors:** Hannah Nicholas, Elinor Winrow, Aisling Devine, Iain Robertson, Ian Mabbett

**Affiliations:** 1 Department of Chemistry, Faculty of Science and Engineering, Swansea University, Swansea, Wales SA2 8PP UK; 2 Specific, Materials Science and Engineering, Swansea University, Swansea, Wales SA2 8PP UK; 3 Department of Biosciences, Faculty of Science and Engineering, Swansea University, Swansea, Wales SA2 8PP UK; 4 Geography Department, Faculty of Science and Engineering, Swansea University, Swansea, Wales SA2 8PP UK

**Keywords:** Faecal sludge, Biochar, Agronomic, Soil, Resource recovery, Sanitation

## Abstract

The disposal of faecal sludge from non-networked sanitation amenities leads to contamination of the surrounding environment and increasing public health problems across developing countries. Permanent solutions to deal with faecal sludge are required to solve the sanitation crisis and achieve the Sustainable Development Goal (SDG) 6 “ensure availability and sustainable management of water and sanitation for all” by 2030. Full-scale pyrolysis of faecal sludge in developing countries is fast becoming a safe and long-term option. Pyrolysis not only eliminates pathogens within the sludge but produces biochar as an end product which has the potential as a soil amendment to increase crop yield. In general, faecal sludge biochars have high pH values, high ash contents, and high macro-and micronutrient concentrations. Compared to biochar from lignocellulosic materials, faecal sludge biochar contains less carbon and exhibits lower porosities, and lower surface areas. However, evaluating the properties of faecal sludge biochar is difficult due to the different technologies used in collection, storage, and transportation of the feedstock. Differences in faecal sludge characteristics based on location, climate, age of the sludge, type of sanitation technology and seasonality are also factors in determining the properties of faecal sludge biochars. These factors contribute to the difficulty in describing faecal sludge biochar properties in general terms, and there is an argument to be made that characteristics of large-scale faecal sludge biochar should be determined on a case-by-case basis. The conclusion of this review is that future research should concentrate on short-term and long-term field studies of faecal sludge biochar application to different soil types.

## Introduction

**Fig. 1:**
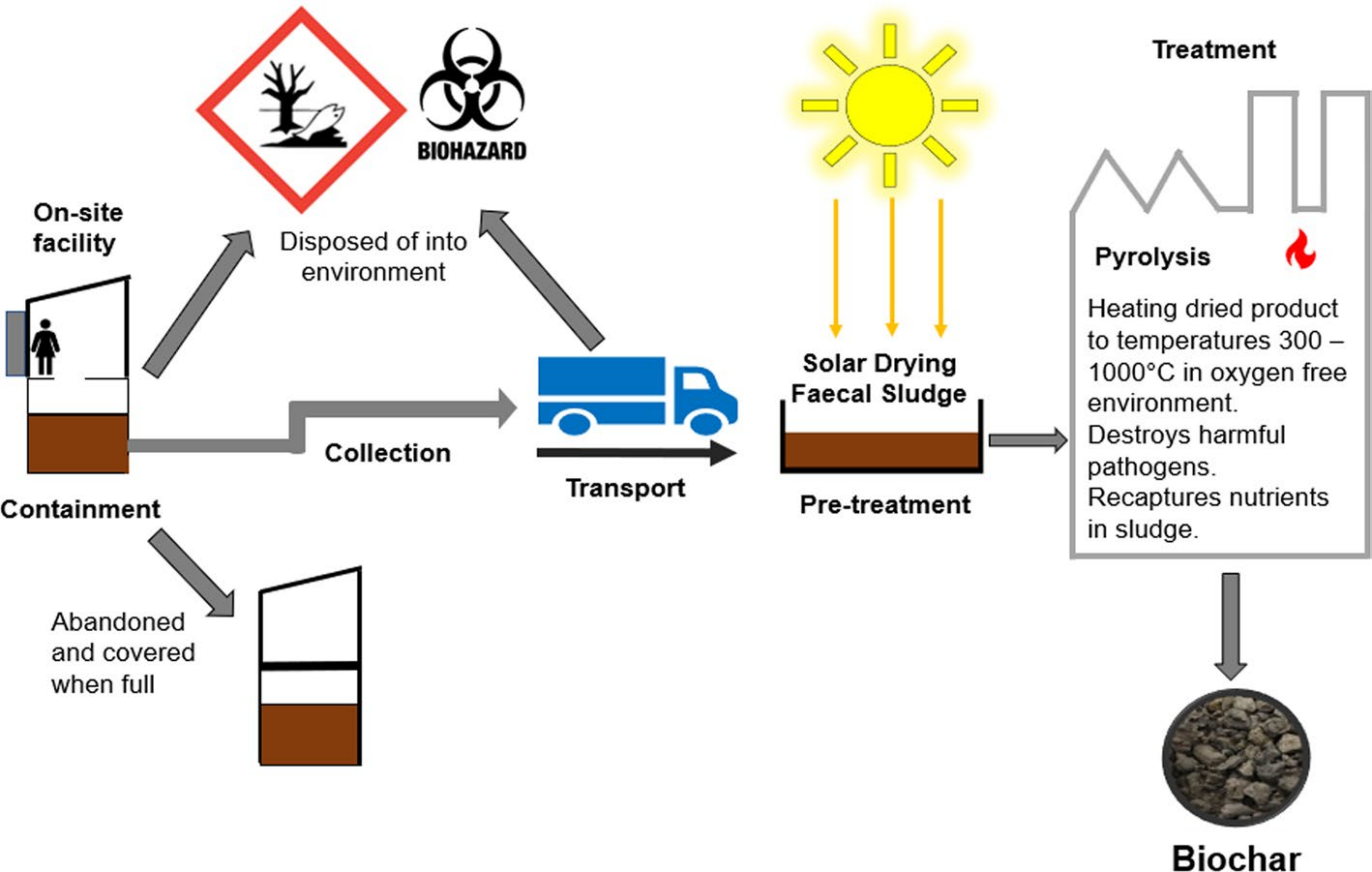
Simplified overview of faecal sludge management with faecal sludge biochar as the end product

- Goal 6 of the UNs 17 Sustainable Development Goals is to “ensure availability and sustainable management of water and sanitation for all” (UN, 2015). In the last 23 years in both low- and middle-income countries, the percentage of the population that utilize “unimproved” sanitation amenities has increased (WHO and UNICEF, 2017). In 2020, 3.6 billion people still lacked access to effectively managed sanitation amenities with 494 million people still practicing open defecation (WHO, 2020). Globally around 2.1–2.6 billion people are reliant on onsite sanitation amenities that produce large quantities of faecal sludge every day (UNICEF and WHO, 2017).
- In developed countries, there are sewer systems and wastewater treatment plants that transport and safely treat sewage sludge. In developing nations, onsite sanitation facilities produce large quantities of faecal sludge (FS). FS is often dumped into the surrounding environment, or reused without any treatment on agricultural land (Jiménez et al., 2009). The poor management of faecal sludge (FS) collected from these onsite sanitation facilities has contributed to worsening public health outcomes and environmental pollution in the form of eutrophication of neighboring lakes and streams, and contamination of soils and groundwater (Gwenzi & Munondo, 2008). These factors contribute to lower economic and social development (Haller et al., 2007; Mara et al., 2010). The focus in developing countries, is on long-term mechanisms to treat faecal sludge generated from non-networked sanitation facilities. However, improving sanitation provision is challenging due to the economic cost as well as the land area, water, and energy requirements. The approach used to deal with these challenges is termed faecal sludge management and is based around 5 main principles which include the storage, collection, transport, treatment and safe disposal of faecal sludge (Strande et al., 2014) (Fig. 1.). Recent research has investigated the thermochemical treatment by pyrolysis as a reliable method of treating faecal sludge. Pyrolysis involves heating biomass to temperatures of 350–1000 °C in the absence of oxygen (European Biochar Foundation, 2016) which thoroughly destroys pathogenic organisms within faecal sludge (Liu et al., 2014b). This process creates a carbon-rich product, biochar, which unlike charcoal does not easily burn and the predominant use of biochar is as a soil amendment (Crombie et al., 2013). Physico-chemical properties of biochar are related to the composition of the original feedstock and the pyrolysis parameters such as holding time and the highest treatment temperature (HTT) (Cairns et al., 2022). This process also yields other by-products including bio-oil, tar and syngas.
- The use of biochar to increase soil fertility and crop yield was introduced as a theory from observations made on Amazonian Black Earth (*terra preta*). This specific type of fertile, very dark, carbon-rich soil, discovered in the Amazon basin, was found to contain greater nutrient content and greater organic carbon content than surrounding soils (Glaser et al., 2001). Biochar as a soil amendment produces many known benefits including improving carbon content and nutrient levels (Glaser et al., 2001), increasing the cation exchange capacity of soils (Glaser et al., 2001), increasing the water-holding capacity of soil (Gaskin et al., 2007; Herath et al., 2013), increasing pH levels in acidic soil (Novak et al., 2009a, 2009b), as well as reducing and immobilizing toxic metals such as arsenic, cadmium and zinc (Park et al., 2011). Biochar application to soil can also provide long-term carbon sequestration, reduce yearly greenhouse emissions and ultimately mitigate climate change (Woolf et al., 2010).
- Far more research has focused on evaluating the benefits of sewage sludge biochar on soil fertility and crop yield (Gwenzi et al., 2016; Hossain et al., 2015; Khan et al., 2013; Liu et al., 2014a; Sousa & Figueiredo, 2016; Tian et al., 2019; Waqas et al., 2015; You et al., 2019; Zhang et al., 2016), compared to faecal sludge biochar (Bai et al., 2018; Woldetsadik et al., 2018).
- The potential benefits of sludge-derived biochar in developing nations are arguably greater than that in developed countries. In developing nations such as in sub-Saharan Africa subsistence farming and small-holder farms are commonplace, however, the soils in these regions are degraded, (Gwenzi et al., 2015). The soils are often of low fertility, low water holding capacity and low pH (Nyamapfene, 1991). There are also constraints to large-scale application of inorganic fertilizer to improve soil fertility such as supply problems, late deliveries, and unsuitable fertilizer blends for the local soil characteristics (Ricker-Gilbert, 2020). These limitations are higher in nations with constrained or non-existent agricultural input subsidy schemes and generally only one-third of Saharan African farmers apply inorganic fertilizers (Sheahan & Barrett, 2017). Developing countries will also see the greatest rise in food demand due to climate change likely devastating crop yields by 15–20% (World Bank, 2015). The re-use of faecal sludge as a biochar soil addition in developing nations would reduce contamination of fresh water sources by untreated faecal sludge disposal. The biochar end product would also reduce fertilizer need, improve soil health and crop yield in areas far more at risk from climate change-induced droughts.
- The composition of biochar is largely dependent on two conditions; the feedstock and the temperature at which the feedstock is pyrolyzed (Downie et al., 2009). Sewage sludge and faecal sludge have different physico-chemical characteristics due to the different transport conditions, treatment processes and holding times. The characteristics of each type of waste can vary significantly, depending on several factors outlined below. In general, human waste is a complex heterogeneous mixture which can contain microorganisms, water, oils, nutrients, inorganic material and can be rich in organic matter.
- Faecal sludge quantities and characteristics can vary greatly depending on several factors including location, climate, age of the sludge, type of sludge collection and the types of onsite sanitation facilities (Strande et al., 2014). These onsite sanitation technologies include septic tanks, aqua privies, pit latrines (including Ventilated Improved Pit Latrines VIPs), public ablution blocks and dry toilets. Another difficulty in quantifying faecal sludge is that in cities different types of these facilities can be found side-by-side.
- This review describes the methods used for the collection and drying of faecal sludge and highlights the properties of faecal sludge and faecal sludge biochar with an emphasis on its end use as a soil amendment.

## Properties of faecal sludge

Biochar properties are strongly influenced by highest heating temperature during pyrolysis and the type of feedstock. Lignocellulosic material produces biochar with markedly different characteristics from that of sewage sludge biochar (Xing et al., 2021). Understanding the properties of faecal sludge is a crucial step in helping to recognize the resultant properties of the biochar end-product.

The pH of faecal sludge generally has a larger range than sewage sludge with pH values of faecal sludge between 6.55 and 9.34 (Kengne et al., 2011) (Table 1). The pH values of faecal sludge have been found to vary between regions. In Ghana, faecal sludge from rural areas was slightly acidic (pH 6.7), whereas sludge from peri-urban areas was found to be alkaline with a pH of 7.3 (Appiah-effah et al., 2014a).

**Table 1:** The physicochemical properties of faecal sludge, total solid content (TS), total volatile solid content (TVS), total suspended solids content (TSS) and ammonium (NH_4_^+^), Ammoniacal nitrogen (NH_3_–N), nitrite–nitrogen (NO_2_–N), total Kjeldahl nitrogen (TKN), potassium (K) and total phosphorus (TP)

References	pH	TS (g/l)	TVS (%)	TSS (mg/l)	NH_4_^+^ (g/l)	NH_3_–N mg/l	NO_2_–N mg/l	TKN (mg/l)	K mg/l	TP mg/l
Appiah-effah et al. (2014a)	6.4–8.1	49,850–84,550 ^a^	75–83	–	–	1890–3875	480–1089	3470–5565	528–1524	879–3831
Kengne et al. (2011)	6.55–9.34	0.3–12.7^b^	31.0–90.7	2.5–124.4^c^	–	–	–	0.3–3.9^c^	–	–
Ward et al. (2019)	6.3–7.9	2.8–34.9	48.3–84.9	1.7–18.3^c^	–	–	–	–	24.4–474.7	–
Piyabalo et al. (2021)	7.3–8.4	–	–	–	337–850.94^e^	–	13.4–17.7^e^	908–4760^e^	–	–
Lama et al. (2022)	7.6	35,506^a^	15,296^a^	28,214	–	157	–	105	178.26	24.21
Bassan et al. (2013)	–	8984–13349^a^	53–61	6826–11,084	1230^a^	–	–	–	–	–
Fanyin-Martin et al. (2017)	7.41–7.87	0.98–4.68^2^	–	–	–	–	–	649.4–4479	–	137.9–521.1
Awere et al. (2020)	–	35.4–36.9^b^	65.5–66.1	–	–	–	–	–	–	–
Schoebitz et al. (2014)	–	5020–71,007^1^	3421–47,440^1^	–	–	–	–	–	–	–
Talla et al. (2017)	7.04	6825^a^	222^a^	16,600	–	–	–	408.84	–	–
Junglen et al. (2020)	4.01–8.30	13.20—116.80	–	4.01–66.70^c^	–	–	–	–	–	–
Gold et al., 2018b)	7.8	1.7–14.8^b^	–	–	–	–	–	–	–	–
Strande et al. (2018)	8.35	25	–	20^c^	–	–	–	–	–	–
Atwijukye et al. (2016)	6.84–9.1	–	–	7455–82,688	–	–	–	755.1–2734^a^	–	176–1181
Heinss et al. (1998)	–	< 3–3.5^b^	–	700–30,000	–	–	–	–	–	–
Forbis-Stokes et al. (2021)	7.7	–	–	296	–	–	–	87.3	–	–
Wanda et al. (2021)	6.96	6.42	–	–	–	–	–	–	–	–

^**a**^mg/l

^**b**^%

^**c**^g/l

^**d**^mgO_2_/l

^**e**^mgN/L

Total solids characterization of faecal sludge is important to be able to design and implement faecal sludge treatment solutions. The total solids present in faecal sludge comprises both organic (vaporizes readily) and inorganic matter. The concentration of faecal sludge total solids have been measured at a range of 12,000–35,000 mg/l (Koné & Strauss, 2004) and volatile solids in faecal sludge measured at 0.45–4.3 g VS/g ash (Zuma et al., 2015). The measurement of total solids is dependent on the moisture content which can be highly variable and give rise to uncertainties when stating different properties based on the total volume (i.e. litre) or mass (i.e. g/ash) (Velkushanova & Strande, 2021).

In raw, untreated wastewater ammonium nitrogen NH_4_–N is the main form of nitrogen, with other forms such as organic nitrogen, nitrite nitrogen (NO_2_−N) and nitrate nitrogen (NO^−^_3_–N) present to a lesser degree (Li et al., 2017). Both ammonium nitrogen and nitrate nitrogen are bioavailable forms for plant uptake and are crucial in evaluating human waste as a soil fertilizer.

Nitrogen in faecal sludge can be found as nitrate, nitrite, organic forms (amino acids) and as ammoniacal-nitrogen with the latter mainly arising from the urine component (Fidjeland, 2015).

Ammoniacal-nitrogen concentration in faecal sludge from septic tanks has been measured at 150–1200 mg/l (Koné & Strauss, 2004) and < 1000 to 2–5000 to mg/l in studies from Ghana, Thailand and Philippines (Heinss et al., 1998). For comparison, a value of 30-70 mg/l for typical municipal sewage in tropical countries was also reported by Heinss et al. (1998). In general, ammoniacal-nitrogen concentration is higher in faecal sludge and septage than sewage sludge.

The levels of nitrates in faecal sludge from septic tanks have been measured at 0.2–21 mg N/L (Koottatep et al., 2005). Total phosphorus levels found in faecal sludge can be very high, it is usually present in phosphate form (e.g., H_3_PO_4_/PO_4_–P) or in the organic phosphate form that is present in plant tissue such as nucleic acids, phosphoproteins and adenosine triphosphate (Niwagaba et al., 2014). The form that phosphorus takes in the faecal sludge depends on various factors such as pH, sedimentation, precipitation and redox potential (Niwagaba et al., 2014).

The different sanitation technologies in use can also affect recoverable nutrient concentrations. Phosphorus and potassium concentrations in sludge from ventilated pit latrines were found to be 3.4 and 3.8 times higher, respectively, than sludge collected from septic tanks, and urine diverting dry toilets contained concentrations of potassium 8.8 times higher than sludge from septic tanks (Krueger et al., 2021).

Human waste contains many different types of pathogens. The pathogens found in faecal sludge are not discussed in this study since the high temperatures (generally > 500 °C) used during pyrolysis destroy the physical structure of pathogens within the sludge (Werther & Ogada, 1999).

Metals of concern that are found in human waste are toxic and harmful to the environment and humans if they enter the food chain. These include cadmium, zinc, nickel, chromium, mercury, lead and copper. Arsenic is often included in this group as it is carcinogenic and a plant toxin (Moreno-Jiménez et al., 2012). Potentially toxic metals in sewage sludge arise from point sources such as households and businesses and diffuse sources such as rainwater runoff from roofs, galvanised materials, traffic and agricultural areas (Bergbäck et al., 2001; Sörme & Lagerkvist, 2002). It is thought that toxic metals are at lower levels in faecal sludge than sewage sludge, with toxic metals in pit latrines found to be at lower concentrations than wastewater sludge (Wang et al., 2021). Toxic metal content of faecal sludge ash has been found to be below the thresholds for land disposal (Barani et al., 2018), however in the same study, it was also discovered that community toilet samples had the highest toxic metal content. The reason suggested for this finding is that community toilets were more likely to be used to dispose of polluting waste. Toxic metals in faecal sludge arise in small quantities from diet and in larger quantities from illegal disposal of hazardous materials such as batteries to latrines (Appiah-Effah et al., 2014b) and leachate infiltration from landfills (Krueger et al., 2020).

Large variations in toxic metal concentrations of faecal sludge depend on factors such as season and location. The concentration of toxic metals in human waste can affect its suitability as a soil amendment/fertilizer.

Data on C, H, N, S and O concentrations in faecal sludge is limited. The elemental composition of faecal sludge (Table 2) shows a relatively low percentage of carbon ranging from 11.39 to 43.69%. FS- and SS-derived biochars generally have low total C concentrations in comparison with cellulose derived biochars (Tomczyk et al., 2020). This is due to the high ash content and low carbon content in the original feedstock of faecal and sewage sludge. The ash content is largely composed of oxides of metals such as potassium, magnesium, iron, silicon and calcium (Hafford et al., 2018) which comes from indigestible nutrients (Rose et al. 2015), digestion during storage in onsite sanitation technologies (Gold et al., 2018a) as well as contamination by sand and grit caused by poorly lined containment structures (Niwagaba et al., 2014a).

**Table 2:** C, H, N, S and O percentages of faecal sludge

Sludge type	C	H	N	O	S	References
Faecal sludge from a septic tank	43.69	6.93	3.48	20.51	–	Bai et al. (2018)
Dried sludge from a septic tank	42.4 ± 1.3	6.9 ± 0.9	5.9 ± 1.0	43.1 ± 3.1	1.7 ± 0.5	Liu et al. (2014b)
Dewatered faecal sludge from pit latrines	34.9 ± 3.3	–	2.4 ± 0.3	–	–	Gold et al. (2018b)
Dewatered faecal sludge	33.83 ± 1.41	–	–	–	–	Manga et al. (2022)
Dewatered faecal sludge	11.39 ± 7.70	–	1.05 ± 1.02	–	–	Cofie et al. (2009)

## Pyrolysis

### Pre-treatment of sludge for pyrolysis

Faecal sludge needs to be dried before pyrolysis can occur, and it represents a critical process within most faecal sludge management systems (FSM). It allows for the removal of moisture, while at the same time, eliminating the pathogen population found within the material and potentially capturing useful by-products such as water and energy (Winrow, 2022). Thermal drying involves the application of heat to a material which results in the transfer of moisture within the material to its surface, and then the removal from the material into the atmosphere (Karathanos & Belessiotis, 1999). It is thought that there are currently around 200 different ways to dry a material with new techniques being developed on a regular basis. This indicates that fully understanding the drying properties of a material are increasingly valuable to ensure that the right technique is chosen (Klemeš et al., 2008).

When looking at the different techniques to use, it is important to consider the composition of the material, its size and shape, the optimum drying temperature, along with the climatic conditions of the environment including humidity and temperature (Karathanos & Belessiotis, 1999; Kipphan, 2001). For faecal sludge, these techniques need to have a low cost, low energy demand and be user friendly.

When a wet solid is subjected to thermal drying, two processes occur simultaneously:

1. Transfer of energy (mostly heat) from the surrounding environment to evaporate the surface moisture (Suryakumar & Pavithra, 2020). The removal of water as vapour from the material surface depends on the external conditions of temperature, air humidity and flow, area of exposed surface, and the pressure.
2. Transfer of internal moisture to the surface of the solid and its subsequent evaporation due to process. The movement of moisture internally within the solid is a function of the physical nature of the solid, the temperature, and its moisture content. 

The rate at which drying is accomplished is governed by the rate at which the two processes proceed, with one process being the limiting factor governing the rate of drying (Mujumdar, 2007).

**Fig. 2:**
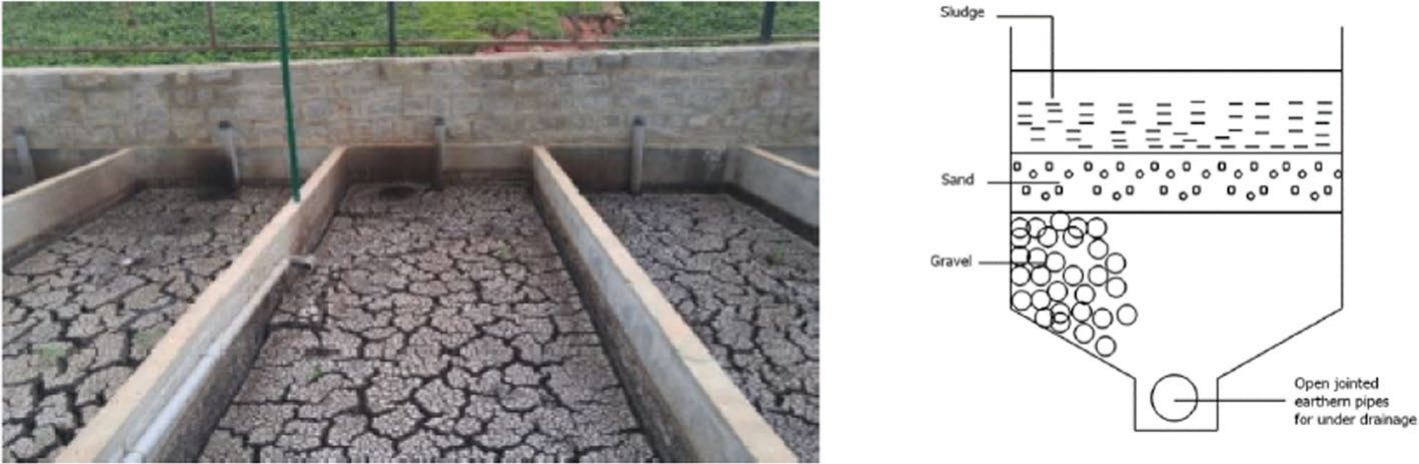
Photo of drying beds the design to efficiently dewater sludge (Bassan et al., 2014; Bhagwat, 2016)

The most common drying technique found within FSM systems are drying beds. This is due to their ease of use, low cost and the ability to remove all pathogens present, provided that the sludge is left in the beds for long enough (Fig. 2).

Drying beds are usually used within small-to-medium-sized communities in low-income countries due to them having low energy requirements, low operating and maintenance costs, and can generate revenue to offset treatment costs through resource recovery as fodder and soil amendments (Gueye et al., 2016; Tchobanoglous et al., 1994). They work by discharging the faecal sludge onto the surface where two different drying processes take place. (1) Dewatering—this is where the water filters through gravel. This process only takes a matter of days if not hours. This is a quick way to remove a large volume of the liquids present which usually lack pathogens. (2) Drying—this is where water leaves through evaporation. This process can take several weeks or months depending on the time of year. Depending on the faecal sludge characteristics, a variable fraction of around 50–80% of the sludge volume drains off as a liquid which then needs to be collected and treated prior to discharge. Once dry, the sludge is mechanically or manually removed from the dry bed for further processing to ensure complete pathogen removal (Bassan et al., 2014) (Fig. 2.).

The pyrolysis process ranges from lab-scale conditions using tube furnaces (Bai et al., 2018), and muffle furnaces (Koetlisi & Muchaonyerwa, 2017) to full-scale conditions (Krueger et al., 2020) (Nicholas et al., 2022). Pyrolysis conditions can vary considerably from highest heating temperatures as low as 300 °C (Liu et al., 2014b) to as high as 750 °C (Krueger et al., 2020) with the range of residence times from 10 min (Gold et al., 2018a) up to 120 min (Koetlisi & Muchaonyerwa, 2017).

Pyrolysis involves the thermal decomposition of carbonaceous material when heated under relatively high temperatures in an oxygen -free environment, producing three main products: bio-oil, combustible gas and biochar (Wei et al., 2022). Pyrolysis can be divided into different classes based on the residence time of the biomass and the operating temperature (Perkins, 2018).

In this review, we focus on the most common method of producing biochar: slow pyrolysis. Slow pyrolysis is defined by slow heating rates between 1 and 30 °C min°1 (Lua et al., 2004) with highest heating temperatures of 400–900 °C in the absence of oxygen. Slow pyrolysis is often deemed the most practical process for agronomic biochar production (Song and Guo, 2012). Slow pyrolysis is generally undertaken at atmospheric pressure, with the process heat supplied from an external energy source. This source can be from combustion of the produced syngas or by partial combustion of the biomass feedstock (Laird et al., 2009).

## Properties of sludge biochar

**Fig. 3:**
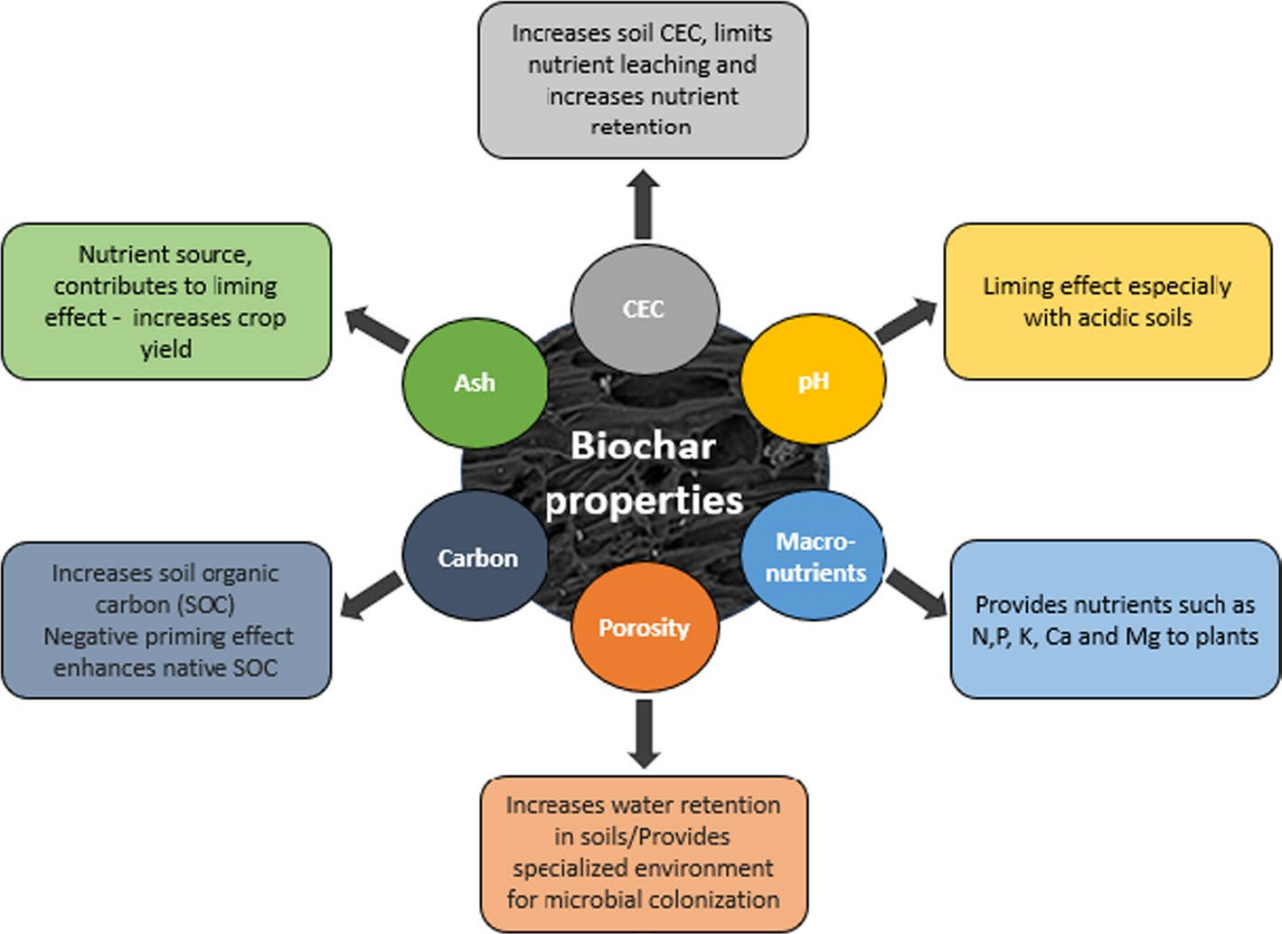
Biochar properties relating to its use a soil amendment (CEC = cation exchange capacity, SOC = soil organic carbon, N (nitrogen), P (phosphorus), K (potassium), Ca (calcium), Mg (magnesium))

Physico-chemical properties and yield of biochar are related to the composition of the original feedstock and the pyrolysis conditions such as the highest treatment temperature (HTT), vapour residence times and heating rate (Kramer et al., 2004). Studies have shown that the HTT is the main parameter in determining final biochar characteristics (Antal & Grønli, 2003; Lua et al., 2004). Properties of biochar that contribute to its use a soil amendment to enhance soil health and increase crop yield include pH, ash content, carbon content, macro-nutrient content, surface area and porosity and cation exchange capacity (Fig. 3).

### pH

The pH of biochar is generally neutral to high and so can increase the pH of soil, this liming effect of biochar can increase plant growth, especially in acidic soils. In fact, the liming effect is one of the main processes influencing the enhanced plant growth seen on biochar addition to soils (Jeffery et al., 2011).

The liming effect can enhance several soil–plant interactions:

- Increase phosphorus availability and N, Ca, Mg and Mo availability
- Reduce the available level of aluminium, which is toxic to plant growth (Hammes and Schmidt, 2009)
- Improvement of N_2_ fixation in legumes
- Enhance microbial activity (DeLuca et al., 2012)

pH conditions can also affect both the adsorption and bioavailability of phosphorus. This effect is particularly evident in acidic soils due to the liming effect of biochar leading to an increase in P availability (Nigussie et al., 2012). The release of nutrients from biochar is also influenced by the pH of the soil, studies have shown an increase in the release of H_*x*_PO_4_ and NH_4_^+^ from biochar with decreasing pH (Silber et al., 2010; Zheng et al., 2013).

Biochar derived from both faecal and sewage sludge generally tend to have high pH values with increasing pyrolysis temperatures leading to an increase in pH (Hossain et al., 2011; Liu et al., 2014b). Examples of pH for faecal sludge biochar are presented in Table 3. It has been proposed that the general alkaline character of biochar results from the carbonate content and the release of alkaline elements such as Na, K, Ca, and Mg during the pyrolysis process (Singh et al., 2010). Altering soil pH is one of several mechanisms by which biochar can improve soils and increase agricultural productivity. Acidic soils are responsible for the severe limitation of crop agriculture worldwide. Up to 50% of soils globally which are suited to arable agriculture are acidic (von Uexküll & Mutert, 1995). Acidic soils are not just responsible for reduced crop yield but also affect the types of crops that can be grown, maize, for example, a staple food crop is adversely affected by acidic soils (Ngoune Tandzi et al., 2018). Faecal sludge biochar has been shown to increase the pH and cation exchange capacity (CEC) of soil (Bai et al., 2018) and application of faecal sludge derived biochar with a high pH to an acidic soil resulted in increased tomato yield, plant height and above ground biomass (Nicholas et al., 2023).

**Table 3:** pH, ash, surface area and CEC (cation exchange capacity) of faecal sludge biochars-hold times are in brackets

Pyrolysis Temperature °C	pH	Ash content %	Surface area (m^2^g^−1^)	CEC cmol_(+)_ kg^−1^	References
600	10.4	–	690.82		Bai et al. (2018)
450	8.23	–	3.36^N2^	23.2	Woldetsadik et al. (2018)
N-BC 500–7001	10.5 ± 0.5	45.6 ± 4.2	–		Krueger et al. (2020)
W_BC 500–700^1^	10.8 ± 1.2	60.8 ± 5.5	–		
350 (10 min)	9.1	54.5	–	9.8	Gold et al., (2018a)
350 (20 min)	9.2(± 0.02)	57.2(± 1.8)	–	13(± 0.7)	
350 (40 min)	9.3	57.5	–	9.8	
450 (10 min)	9.7	65.6	–	22.9	
450 (20 min)	9.7(± 0.02)	66.9(± 1)	–	23.2(± 0.9)	
450 (40 min)	9.7	66.2	–	23.5	
600 (10 min)	11	68.1	–	24.6	
600 (20 min)	11.1(± 0.01)	72.9(± 0.9)	–	26(± 1.7)	
600 (40 min)	11.2	73.8		27.7	
BC-300	7.3 ± 0.1	26.3 ± 0.8	–		Liu et al. (2014b)
BC-400	7.5 ± 0.1	31.3 ± 0.9	–		
BC-500	10.3 ± 0.2	45.5 ± 1.2	–		
BC-600	10.7 ± 0.2	58.8 ± 0.6	–		
BC-700	11.1 ± 0.2	62.5 ± 0.4	–		
350	6.94	84.6	7.5	5.09	Koetlisi and Muchaonyerwa (2017)
550	7.02	90.23	23.7	4.91	
650	7.14	92.97	25.7	5.65	
WAI_BC 550–750 °C	11.81 ± 0.01	62.3 ± 0.32	3.52 ± 0.78	90.0 ± 6.5	Nicholas et al. (2022)
NSP_BC 550–750 °C	11.82 ± 0.01	67.0 ± 2.68	3.69 ± 0.36	41.9 ± 2.2	
WGL_BC550–750 °C	12.45 ± 0.01	88.3 ± 0.21	12.07 ± 4.12	129.3 ± 2.3	

^1^FS was co-treated with pellet fuel derived from agricultural waste (0.3 kg PF/kg FS dry basis)

### Ash

A high ash content is a positive when viewing the applicability of biochar as a soil amendment as the soil benefits from the minerals such as calcium carbonate, silicates and potassium found in ash. Ash on its own has been shown to increase maize yield by eight times greater than plants in a control group and was also found to provide the most nutrients when compared to other soil additions such as lime and biochar (Hale et al., 2020). Rice-husk biochar also contains fairly high ash content and is thought to provide more calcium carbonate and potassium to the soil, allowing more binding surfaces to hold cations (Asirifi et al., 2023). It is accepted that the concentration of ash in biochar is generally higher than in the original feedstock regardless of pyrolysis temperature. Furthermore, an increase in pyrolysis temperature leads to an increase in the ash content of biochar (Fuertes et al., 2010). Ash content also differs greatly depending on the feedstock used. Poultry litter biochar has been described as having an ash content of 30.7% (Cantrell et al., 2012) compared to pine wood chip biochar of only 1.5% (Spokas et al., 2012) with both pyrolyzed at 350 °C. The initial feedstock of faecal sludge is high in ash and has been measured at 17.0 wt%, significantly higher than measured ash content of sawdust at 0.8% (Liu et al., 2014b). It is thought that digestion during storage in onsite sanitation technologies can also play a part in the high ash content (Gold et al., 2018a) of faecal sludge biochar as well as contamination of faecal sludge by sand and grit caused by poorly lined containment structures (Niwagaba et al., 2014), as well as sand adhered to the faecal sludge from the surface of drying beds (Cunningham et al., 2016). Recently, a study comparing mixed urine and faeces (MUF) biochar and source-separated faeces (SFF) biochar found that MUF biochar exhibited higher ash contents which was associated with greater quantity of inorganic salts in urine (Koulouri et al., 2023). The high ash content of faecal sludge biochar is related to the pH values with increasing pyrolysis temperatures leading to an increase in pH due to an increase in ash in biochar. Faecal sludge biochars with ash contents ranging from 62 to 88% produced greater tomato yield compared to control and fertilizer when added to acidic soil (Nicholas et al., 2023).

### Surface area and porosity

The porous structure of biochars resembles the cellular structure of the original feedstock (Fuertes et al., 2010; Yao et al., 2011). In faecal sludge, these cellular macroporous structures arise from undigested fibrous vegetable matter. This porous structure can provide a specialized environment for the colonization of microbes (Thies & Rillig, 2012). An increase in mycorrhizal fungi lead to an increase in mineralization of recalcitrant soil organic matter, thus improving soil and plant health (Anderson et al., 2011; Zimmerman et al., 2011). Water retention of soil is also greatly improved by the addition of biochar. Water retention in *terra preta* was found to be 18% greater than in adjacent soils that contained little or no charcoal (Glaser et al., 2002). One of the benefits of biochar is its recalcitrant nature making it generally stable in soil thus the benefits can be long-lasting. Biochar itself has highly variable water-holding capacity and can even hold more than 10 × own weight in water (Kinney et al., 2012) due to its porous nature with its large specific surface area. The porous structure of biochar results in greater water holding capacity of soil (Herath et al., 2013) and increases water availability (Blanco-Canqui, 2017; Omondi et al., 2016; Uzoma et al., 2011).

**Fig. 4:**
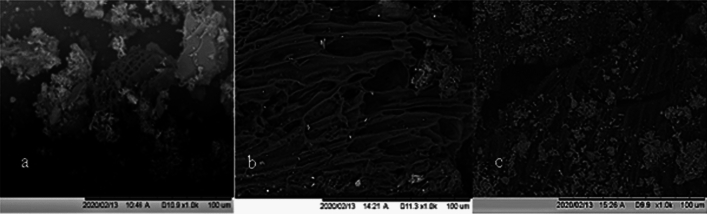
SEM micrograph of faecal sludge biochars with ash particles in white clearly visible (Nicholas et al., 2022)

Increasing the pyrolysis temperature can enhance the BET surface area with more pores within the structure due to an increase in volatile matter released. The fast pyrolysis of municipal sludge biochar at temperatures 500–900 °C showed that increasing temperatures resulted in a greater yield of biochar and greater microporous network within the biochar (Chen et al., 2014). Biochar derived from wastes biological wastes such as manure and faecal sludge generally exhibit lower surface areas than that from lignocellulosic biochars. It is thought that high ash contents reduce surface area by filling or blocking access to the biochar micropores (Song and Guo, 2012). SEM images of faecal sludge biochar show a honeycomb-like structure with cylindrical and slit like holes in biochar particles (black) and a high presence of clay mineral particles/ash(white/grey) (Fig. 4). Surface areas of faecal sludge biochars have been reported at 3.7 m^2^g^−1^, 25.7 m^2^g^−1^ and between 3.5 and 12.1 m^2^g^−1^ (Koetlisi & Muchaonyerwa, 2017; Nicholas et al., 2022; Woldetsadik et al., 2018;) (Table 3). Greater surface areas of faecal sludge biochar (690.8 m^2^g^−1^) have been attained by measuring biochar < 74 µm and demineralizing with 2 M HCl (Bai et al., 2018).

### Cation exchange capacity (CEC)

Biochars unique and varied surface chemistry plays a key role in nutrient leaching and retention in soils. Biochar is negatively charged, thus contributing to electrostatic adsorption of cations (Hale et al., 2013; Yao et al., 2011). The oxygen containing functional groups present on biochars surface such as C=O groups determine its cation exchange capacity (CEC). (Yuan and Xu, 2012). It is this property that enables biochars to adsorb cationic nutrients such as NH^4+^, Ca^2+^, K^+^. This characteristic of biochar results predominantly from formation of carboxylic functional groups during oxidation (Cheng et al., 2006). These surface functional groups on the surface of biochar can lead to an increase in the CEC of the soil (Glaser et al., 2001). CEC is an indicator of a soil’s nutrients-holding capacity and thus soils with high CEC values are generally fertile. The high CEC of biochars combined with large surface areas contribute to limit nutrient leaching in soils, (Lehmann & Joseph, 2012) and improves nutrient retention (Song and Guo, 2012). Addition of biochar to soil has shown increases in cation exchange capacity (CEC) and pH leading to its use as soil amendment (Bai et al., 2018; Glaser et al., 2001).

Due to the porous structure and alkaline ashes present in biochar, the determination of the cation exchange capacity (CEC) is challenging. Reported values for the CEC of biochar are surprisingly variable and are often poorly reproducible, suggesting methodological problems (Munera-Echeverri et al., 2018). CEC values for biochar can range from 6 cmol_(+)_ Kg^−1^ (Munera-Echeverri et al., 2018) to 36.3 cmol_(+)_ Kg^−1^ (Song and Guo, 2012) to as high as 304 cmol_(+)_ Kg^−1^ (Yuan et al., 2011). CEC values of faecal sludge biochar have been reported at 23.2 cmol_(+)_ kg^−1^ for biochar pyrolyzed at 450 °C (Woldetsadik et al., 2018) and 129 cmol_(+)_ kg^−1^ for biochar pyrolyzed at between 550 and 750 °C (Nicholas et al., 2022).

Due to the lack of CEC values in the literature for faecal sludge biochar, it is difficult to draw any conclusions about the affect feedstock and pyrolysis temperature has on CEC values. Previous research has shown inconsistent findings with CEC values of wood char decreasing with pyrolysis temperature (Crombie et al., 2015), but increasing with pyrolysis temperature for cow manure char (Hossain et al., 2011) up to a pyrolysis temperature of 500–550 °C, with a decrease above these pyrolysis temperatures. Gold et al., (2018a) demonstrated that CEC value of faecal sludge char increased with pyrolysis temperature up to a temperature of 600 °C. A study by Koetlisi and Muchaonyerwa (2017) determined CEC values of biochar derived from faecal sludge (latrine waste). They reported a decrease in CEC values for both biochars with increasing pyrolysis temperature from 350 to 550 °C, however CEC values of biochars increased when pyrolysis temperatures were increased to 650 °C. The reported CEC for these biochars are also very low, even lower than the CEC values of the original feedstock (11.7–17.8 cmol _(+)_ kg^–1^). Faecal sludge biochar saturated with ammonium has been shown to increase the CEC of soil from 8.4 cmol/kg (weak nutrient retention and supply capacity) to 13.6 cmol/kg (medium nutrient retention and supply capacity) after 140 days (Bai et al., 2018). Examples of CEC values for faecal sludge biochars are given in Table 3.

### Elemental microanalysis (C, H, N, S and O)

The recalcitrant nature of biochar leads to a build-up of soil organic carbon (SOC) upon biochar addition to soil and has been shown to significantly increase the total organic carbon content of soil (Dong et al., 2018). A meta-analysis of 50 research papers showed biochar addition significantly enhanced SOC content by 40% (Liu et al., 2016). Biochar can also increase organic matter in soil by providing micro-organisms with carbon from the labile component of biochar and/or by the priming effect for the loss of soil organic matter (SOM). There are contradicting positive and negative effects of biochar application on SOM fractions. Meta analyses conducted by Joseph et al., (2021) found that biochar builds soil organic carbon via negative priming by 3.8%. There is limited data available on the effect of faecal sludge biochar on carbon mineralisation in soil. SS biochar addition to soil has resulted in increased soil organic carbon in acidic, paddy soil (Khan et al., 2013) and dairy manure biochar has effected the soil labile carbon pool along with microbial community structures in depositional and eroded landscape positions (Sandhu et al., 2019). Labile C addition from biochar also increases immobilisation of N and is a method by which biochar-amended soil shows increased N retention and reduced N leaching (Clough et al., 2013).

Faecal sludge and sewage sludge—derived biochars generally have low total C concentrations (8–40%) in comparison with cellulose derived biochars (Tomczyk et al., 2020). This is due to the high ash content in the original feedstock. A higher ash content in the feedstock indicates a lower carbon content in the final biochar. Pyrolysis generally concentrates carbon in the biochar with an increase in C content relative to the feedstock frequently reported, however most studies on sewage sludge –derived biochar show a decrease in the percentage of C in the final product relative to the feedstock (Agrafioti et al., 2013; Khan et al., 2013). An increase in pyrolysis temperature leads to a decrease in C and N and an increase in the ash content suggesting that as more ash is relatively accumulated, C and N are reduced. Examples of carbon content in faecal sludge biochars is given in Table 4.

**Table 4:** Organic components, C, H, N, O and H/C ratios of selected faecal sludge biochars at different pyrolysis temperatures with holding times in brackets

Pyrolysis temperature °C	C (%)	H (%)	O (%)	N (%)	H/C	References
600	84.37	2.4		0.77		Bai et al. (2018)
450	19.5			2.02		Woldetsadik et al. (2018)
N-BC 500–700^1^	34.1 ± 3.9	–	–	–	–	Krueger et al. (2020)
W_BC 500–700^1^	17.2 ± 5.2	–	–	–	–	
350 (10 min)	33.3(± 2.7)	–	–	–	–	Gold et al.(2018a)
350 (20 min)	33.5(± 2.4)	–	–	2.3(± 0.0)	–	
350 (40 min)	34.9(± 2.8)	–	–	2.3(± 0.0)	–	
450 (10 min)	32.8(± 1.2)	–	–	2.0(± 0.0)	–	
450 (20 min)	27.4(± 4.1)	–	–	1.6(± 0.0)	–	
450 (40 min)	31.5(± 4.1)	–	–	1.8(± 0.0)	–	
600 (10 min)	29.8(± 2.3)	–	–	1.5(± 0.0)	–	
600 (20 min)	28.2(± 2.2)	–	–	1.3(± 0.0)	–	
600 (40 min)	27.4(± 2.7)	–	–	1.3(± 0.0)	–	
BC-300	42.9 ± 1.2	6.7 ± 1.4	44.4 ± 1.9	4.8 ± 0.6	1.88	Liu et al. (2014b)
BC-400	42.0 ± 2.1	3.5 ± 0.4	50.1 ± 1.7	3.4 ± 0.6	0.99	
BC-500	35.7 ± 3.9	1.9 ± 0.4	58.4 ± 3.3	2.9 ± 0.7	0.62	
BC-600	37.9 ± 1.3	1.8 ± 0.2	56.4 ± 1.2	2.9 ± 0.9	0.56	
BC-700	36.4 ± 4.3	1.8 ± 0.8	58.3 ± 2.3	2.4 ± 0.8	0.58	
350	11.14	1.01	–	1.04	1.1	Koetlisi and Muchaonyerwa (2017)
550	8.73	0.36	–	0.71	0.3	
650	6.45	0.35	–	0.44	0.4	

^1^FS was co-treated with pellet fuel (PF) derived from agricultural waste (0.3 kg PF/kg FS dry basis)

High concentrations of N indicate biochar can be used directly as a fertilizer (Chang et al., 2015) and a review by Clough et al.,(2013) supports the theory that manure derived biochars can play a role as nitrogen-based fertilizers. Yield increases have been seen upon addition of high nitrogen content biochar such as poultry litter biochar (Spokas et al., 2012), and manure derived biochar has shown increased yields as well increased plant nitrogen uptake (Uzoma et al., 2011). Biochar N can be decomposed in the soil and provide nitrogen to plants (de la Rosa & Knicker, 2011) and isotopic labelling of slurry derived biochar –N revealed that biochar-N was utilized by plants. Total nitrogen content in biochars can vary considerably across a large range (Bridle & Pritchard, 2004), with total nitrogen content of sewage sludge biochars reported as higher than biochars produced from green wastes. It is thought the nitrogen exits in faecal sludge in mainly organic forms (Tian et al., 2013) and is volatilized at temperatures around 200 °C (DeLuca et al., 2012), and thus, the actual N content can be very low. Hossain et al. (2011) reported N content in wastewater sludge biochars increased from between 1.2 to 3.32% with decreasing pyrolysis temperature and in faecal sludge biochar nitrogen content has been reported at 0.37% (Nicholas et al., 2022) and 4.8% (Liu et al., 2014b).

### Potentially toxic metals

The toxic metal concentration is highly variable both in sewage and faecal sludge and this impacts the toxic metal content in the biochar. Studies have shown that in biochars containing high concentrations of potentially toxic metals the pyrolysis process entraps the metals in immobile and stable forms within the biochar (Galvín et al., 2009; Sun et al., 2018; Wang et al., 2016). Toxic metal content of faecal sludge biochar has been found to be lower than in sewage sludge biochar (Bleuler et al., 2021; Gold et al., 2017). Toxic metal concentration in biochars generally increase with pyrolytic temperature (Lu et al., 2013). Toxic metals in faecal sludge-biochar adhere to the general trend with an increase in toxic metal concentrations with an increase in pyrolysis temperature (Gold et al., 2018a) (Table 6). Biochars pyrolyzed at higher temperatures can have beneficial qualities for use as a soil amendment including higher pH values and greater surface areas. Toxic metals in most faecal sludge derived biochars are below International Biochar Initiative (IBI) accepted upper thresholds (IBI, 2015). Comparison of potentially toxic metal thresholds are given in Table 5. One exception to this is faecal sludge derived biochar studied by Woldetsadik et al., 2018. This biochar pyrolyzed at 450 °C contained Zinc and Pb in excess of the upper thresholds (IBI, 2015). No explanation for this was given but toxic metal concentrations in faecal sludge can vary considerably depending on season and location. Potentially toxic metal thresholds are given in Table 5

**Table 5:** Comparison of potentially toxic metal thresholds (European Biochar Foundation (EBC), 2016; IBI, 2015)

European biochar certificate V4.8	IBI biochar standards
Basic grade	V2.0 B maximum allowed thresholds
(mg kg^−1^)	(mg kg^−1^)
Cd < 1.5	Cd 1.4–39
Ni < 50	Ni–600
Cu < 100	Cu 63–1500
Hg < 1	Hg 1–17
Cr < 90	Cr 64–1200
–	Co 40–150
–	Mo 5–20 mg
–	Se 2–36
–	As 12–100

Studies looking at faecal sludge biochar have also investigated the potential leaching of toxic metals from biochar.

The soluble and extractable fractions of toxic metals in biochar is significantly decreased when compared to the original sludge feedstocks and the total toxic metal concentrations in biochar (Sun et al., 2018).

There have been several reasons suggested for this trend:

- Amines and amides remaining at pyrolysis temperatures > 300 °C behave as ligands for binding potentially toxic metals in the sludge and entraining the metals within the carbon structure network (Jin et al., 2014)
- High phosphorus content can stabilize toxic metals through the formation of an insoluble phosphate precipitant (Lu et al., 2013)
- High pH values (commonly found in sewage sludge and faecal sludge chars) tend to restrain toxic metal release (Kistler et al., 1987)

Hossain et al.(2011) showed that DTPA-extractable concentrations of potentially toxic metals decreased with increasing pyrolysis temperature from 300 to 700 °C, however in another study, extractable toxic metal concentrations in sewage biochar increased with pyrolysis temperature in the range 300–500°C (Lu et al., 2013). There is limited research available on the effect of pyrolysis temperature on extractable toxic metal concentrations in faecal sludge. Potentially toxic metal concentrations of selected faecal sludge biochars are given in Table 6.

**Table 6:** Potentially toxic metal concentrations of selected faecal sludge biochars

Pyrolysis temperature °C	g kg^−1^	mg kg^−1^					References
	Zn	Cd	Ni	Cr	Pb	Cu	
450	28.4	1.23	84.4	39.5	502	214	Woldetsadik et al. (2018)
N-BC 500-700^1^	1. 5 ± 0.2	13.5 ± 2.7	122.7 ± 37.1	6.1 ± 5.8	395.3 ± 57.9	463.0 ± 61.1	Krueger et al. (2020)
W_BC 500-700^1^	1.1 ± 0.3	12.4 ± 2.0	164.1 ± 48.8	54.3 ± 16.5	241.7 ± 50.9	310.3 ± 37.0	
350 (10 min)	0.917 ± 0.05	–	63.7 ± 2.7	121.5 ± 7.1	< 5	90.4 ± 3.9	Gold et al.(2018a)
350 (20 min)	0.923 ± 0.06	–	63.3 ± 5	124.9 ± 8.8	21.5	86.6 ± 9.4	
350 (40 min)	0.873 ± 0.005	–	57.4 ± 0.9	113.7 ± 4.0	< 5	81.8 ± 1.9	
450 (10 min)	0.971 ± 0.06	–	62.9 ± 3.8	125.2 ± 4.6	14.9 ± 1.4	96.7 ± 9.2	
450 (20 min)	1.01 ± 0.01	–	66.6 ± 1.9	129 ± 3.5	13.7 ± 2.4	101.7 ± 1.6	
450 (40 min)	0.948 ± 0.01	–	76 ± 1.9	152.6 ± 3.5	< 5	91.5 ± 1.6	
600 (10 min)	1.06 ± 0.02	–	89.8 ± 3.1	180 ± 4.8	< 5	101.6 ± 4.3	
600 (20 min)	1.09 ± 0.08	–	78 ± 3.5	151.9 ± 6.1	< 5	110. ± 8.0	
600 (40 min)	1.12 ± 0.037	–	96.5 ± 3.2	194.2 ± 6.7	< 5	113.2 ± 7.9	

^1^FS was co-treated with pellet fuel (PF) derived from agricultural waste (0.3 kg PF/kg FS dry basis)

### Phosphorus

Faecal sludges are rich in mineral nutrients such as ammonium, nitrate, potassium, trace elements and phosphate, the latter of which is a finite resource and an irreplaceable plant limiting nutrient (Steen, 1998). Reported concentrations of phosphorus on a dry weight basis in sewage sludge can range from < 0.1 to 14% (Sommers, 1977). The phosphorus concentration in biochar is increased relative to the original feedstock due to volatilization of elements C, H, O and N during pyrolysis (Sousa & Figueiredo, 2016). The general trend observed with total phosphorus and pyrolysis temperature is increasing phosphorus content with increasing temperature. Chan and Xu, (2009) reported an increase in phosphorous from 5.6% at 250 °C to 12.8% at 800 °C in sewage sludge biochar.

It is thought that phosphorus within sewage sludge is mainly in inorganic form therefore is more susceptible to volatilization losses specifically at pyrolysis temperatures over 700 °C (Gaskin et al., 2008). This effect has been recorded in studies of faecal sludge biochar (Gold et al., 2018a; Liu et al., 2014b), however at pyrolysis temperatures of 700 °C Liu et al., (2014b) recorded a decrease in phosphorus content in faecal sludge biochar and Zielińska et al. (2015) observed an increase of P content in sewage sludge biochar. The conflicting trend of phosphorus content at pyrolysis temperatures of 700 °C may be caused by variations in the forms of phosphorus present in different types of sludge. Both the composition of raw sludge and differing chemical and biological treatment processes can alter the forms of *P* present (McLaughlin, 1984), and hence alter the resistance to volatilization losses at temperatures > 700 °C. Gold et al., (2018a) reported an increase in Total P concentration in faecal sludge biochar with P content increasing from 3.2% at 350 °C to 3.9% at 600 °C and Liu et al., (2014b) reported an increase from 5.4 at 300 °C to 8.1 wt.% at 600 °C and then a slight decrease at 700 °C.

Not all phosphorous within biochar is available to plants, the phosphorus available to plants within biochar is less than the total phosphorus in biochar. Pyrolysis of sludge does increase the amount of available phosphorus within biochar relative to original sludge feedstock (Liu et al., 2014b), in fact, Barry et al. (2019) state that the availability of phosphorous within biochar amended soils is the most significant impact of sewage sludge biochar application. Biochar-added soils have much higher organic available *P* compared to soil without biochar amendment but mechanisms leading to the release of nutrients from biochar are still not fully understood. Added nutrients from the biochar itself is one cause however there are other mechanisms such an increased nutrient retention capacity from the biochar (Joseph et al., 2018) and also the liming effect of biochar which improves nutrient use efficiency and enhances the plant-available *P* in soils (Chintala et al., 2014; Glaser & Lehr, 2019). Hossain et al. (2011) reported that available phosphorus (Colwell P-method) decreased with the temperature from 400 to 700 °C, and Tian et al.(2019) observed a decrease in extractable P from pyrolysis temperatures 200–700 °C. A study of faecal sludge biochar showed the opposite trend was true with an increase in available *P* from 26.1 g/kg at 350 °C to 33.3 g/kg at 600 °C (Gold et al., 2018a) (Table 7).

**Table 7:** Total (Total P) and extractable phosphorus (Available P) content of faecal sludge biochars

Pyrolysis Temperature °C	Total *P* g/kg	Available *P* g/kg	References
450	42.7	–	Woldetsadik et al. (2018)
N-BC 500-700^1^	1.2 ± 0.2	61.0% ± 6.4	Krueger et al. (2020)
W_BC 500-700^1^	2.2 ± 0.6	53.7% ± 12.1	
350 (10 min)	3.2 (± 0.2)	26.1	Gold et al. (2018a)
350 (20 min)	3.3 (± 0.3)	25.3(± 1.4)	
350 (40 min)	3.1 (± 0.0)	28.1	
450 (10 min)	3.6 (± 0.2)	32.6	
450 (20 min)	3.8(± 0.1)	30.5 (± 0.7)	
450 (40 min)	3.5(± 0.1)	34.8	
600 (10 min)	3.9(± 0.2)	33.3	
600 (20 min)	4.0(± 0.2)	33.4(± 1.4)	
600 (40 min	4.2(± 0.3)	36	
BC-300	5.4 wt.% ± 1.2	–	Liu et al. (2014b)
BC-400	6.3 wt.% ± 3.1	–	
BC-500	7.9 wt.% ± 1.7	–	
BC-600	8.1 wt.% ± 1.6	–	
BC-700	7.8 wt.% ± 2.2	–	

^1^FS was co-treated with pellet fuel (PF) derived from agricultural waste (0.3 kg PF/kg FS dry basis)

Yuan et al. (2016) found that sewage sludge biochar has potential as an efficient slow-release phosphate fertilizer to maintain soil fertility long term. In biochar from faecal sludge from dry toilets 65% dry mass of the total phosphorous was found to be plant available, higher than that in biochars derived from wastewater sludge (Bleuler et al., 2021). Sewage sludge biochar addition to soil has resulted in increased phosphorus in soil and increased radish yield (Sousa & Figueiredo, 2016). Biochar produced from source separated faeces and mixed urine faces was determined to have good P recovery potential due to the presence of phosphate compounds of high fertilizer value (Koulouri et al., 2023).

### Macronutrient concentrations (Ca, Mg, K)

Macronutrients are essential for plant growth and development. Calcium is required by plants for cell wall and membrane stability, and also serves as a second messenger in the response of plants to biotic stress (Thor, 2019). Magnesium is necessary for many plant functions including root formation and photosynthesis (Cakmak & Yazici, 2010). As well as playing a significant role in plant growth, Potassium (K) aids with the cotransport of sugars and increases plants abiotic stress tolerance (Johnson et al., 2022). Gondek et al. (2019) found that the high amounts of K, Mg, and Ca found in biochar are favourable for plant growth. Peanut shell derived biochar increased the supply levels of calcium, potassium and magnesium and resulted in increased root and shoot biomass of maize crop (Yang et al., 2020). Biochar treatment has also been shown to enhance tomato growth under saline water irrigation due to the release of macronutrients Ca, Mg and K into soils (She et al., 2018).

Faecal sludge biochars contain significant amounts of macro-nutrients with pyrolysis increasing the concentrations of these elements in biochar relative to the sludge. Increases in Ca, K, and Mg have also been identified with increases in pyrolysis temperature. This is caused by to the gradual loss of C, H and O whereas elements Ca, K and Mg, cannot be lost through volatilization, since the oxides of these metals are not volatile (Al-Wabel et al., 2013). Evidence of large amounts of Ca, Mg and K in faecal sludge biochar has been reported previously (Krueger et al., 2020; Woldetsadik et al., 2018). Evidence of increasing Ca, Mg, and K concentrations with increasing pyrolysis temperature in faecal sludge derived biochar has been reported by Liu et al., (2014b). Macronutrient concentrations of faecal sludge biochars are given in Table 8.

**Table 8:** Macronutrient concentrations (Ca, Mg, K) in faecal sludge biochars

Pyrolysis temperature °C	K	Mg	Ca	References
450	28.9	–	32.8	Woldetsadik et al. (2018)
N-BC 500**-**700^1^	8.1 ± 0.8	7.8 ± 0.7	56.4 ± 3.9	Krueger et al. (2020)
W_BC 500**-**700^1^	11.7 ± 1.9	9.6 ± 1.7	89.4 ± 11.5	
BC-300	1.9 ± 0.9^2^	–	–	Liu et al. (2014b)
BC-400	2.1 ± 0.9^2^	–	–	
BC-500	2.8 ± 0.3^2^	–	–	
BC-600	2.7 ± 0.9^2^	–	–	
BC-700	2.6 ± 0.6^2^	–	–	

^1^FS was co-treated with pellet fuel (PF) derived from agricultural waste (0.3 kg PF/kg FS dry basis)

^2^= wt%

Values in g/kg unless otherwise stated

### Micronutrients

Micronutrients, Zinc (Zn), copper (Cu), iron (Fe), manganese (Mn), boron (B), molybdenum (Mo), chlorine (Cl) and nickel (Ni) play a significant role in the growth and development of plants. Biochar application to soil has been shown to enhance soil fertility by increasing micronutrient levels in soil. In particular the ash component of biochar contains significant amounts of micronutrients which upon addition to soil can impact the micronutrient levels in soil and increase soil fertility (Chan & Xu, 2009; Shaaban et al., 2018). In general, there is not a great deal of literature investigating the mechanisms and extent to which biochar can provide micronutrients to enhance plant growth in soils. Only Fe, Zn, and Cu contents of biochar are usually reported (Hossain et al., 2020) and often time micronutrients are often referred to as *other nutrients* in the literature. Faecal sludges contain relatively large amounts of micro-nutrients that can contribute to enhanced soil fertility. Faecal sludge derived biochars therefore also generally have a relatively high concentration of micronutrients and similarly to macro-nutrients, pyrolysis increases concentrations of these elements within the biochar. Woldetsadik et al. (2018) measured iron concentration (24.4 g/kg) in faecal sludge biochar, Krueger et al. (2020) measured zinc concentration at 1516.9 mg/kg ± 209.1, nickel concentration at 164.1mg/kg ± 48.8, and copper concentration at 463.0 mg/kg ± 61.1 in faecal sludge biochar.

Silicon is not considered as an essential nutrient for plants, but it is believed to be a beneficial element for many plants (Epstein, 1994), in silicophilic plants Si is a major nutrient element. X-ray crystal diffraction of sewage sludge shows that SiO_2_ is a major contributor to sewage sludge biochars with SiO_2_ ranging from 35.8 to 58.1% of all crystallographic structures (Zielińska et al., 2015). They concluded that the presence of SiO_2_ in the sludges is related to the sand removed from sewage as a result of mechanical pre-treatment. However, both sewage and faecal sludge have high mineral components not dependent on the treatment processes and this high mineral content is still evident as faecal sludge biochars record high silicon, iron, sodium and manganese concentrations. X-ray crystal diffraction of faecal sludge biochar also indicated a strong presence of SiO_2_ within the biochar (Nicholas et al., 2022). Rice husk biochar is known to contain high silica concentrations which improves crop growth, reduces abiotic stress, promotes photosynthesis and enhances plant resistance against disease (Singh Karam et al., 2022). There are very few studies that have investigated the Si concentration in FS biochar and its role in soil and increased plant growth. There is potential for further research on this topic, especially the role that silicon in faecal sludge biochars can play in alleviating plant stress.

#### Effect of faecal sludge biochar on crops

There are very few studies that investigate faecal sludge biochar as a soil amendment (Nicholas et al. (2023). The addition of faecal sludge biochar to an structurally stable tree (SST) substrate can significantly improve conditions for tree seedlings in terms of tree survival (Saluz et al., 2022); a finding the authors concluded was due to faecal sludge biochar proving nutrients and increasing the substrate's water storage capacity. Woldetsadik et al. (2018) found that FS biochar increased yield and nutrient concentration of lettuce in two contrasting soils. Of note was the finding that FS biochar addition increased yield to a greater extent in a less fertile sandy loam soil. Above ground biomass was also significantly increased for both soils at the biochar application rates of 20 ha^−1^. They suggest that faecal sludge biochar could be used as an effective fertilizer to increase lettuce yields in low fertility, sandy loam and moderately fertile silty loam soils. The nutrient concentration of sewage sludge biochars has led to the idea that sludge biochar can be an effective fertilizer (Kahiluoto et al., 2015).

Biochar saturated with ammonium has been investigated as a conditioner and was found to increase soil CEC and soil pH (Bai et al., 2018). More recently faecal sludge biochar has been shown to increase the plant height, below ground biomass and yield of tomatoes grown in acidic soil (Nicholas et al., 2023). This study also showed that FS biochar addition increased above ground biomass compared to control and fertilizer treatment alone, a similar finding to the study by Woldetsadik et al. (2018). These findings give credence to the idea that faecal sludge biochar could be used as an effective fertilizer and not just a soil conditioner. A meta-analysis by Ye et al. (2020) concluded that biochar and inorganic fertilizer addition causes an increase in yield ≥ 15% greater than fertilizer treatment without any biochar amendment. The main reason for the increases in yield is thought to be related to the liming effect from alkaline biochar.

## Conclusion

This review is the first to summarize properties of faecal sludge biochar with a view to its end-use as soil amendment.

Potentially toxic metals are generally found at lower levels in faecal sludge biochars than sewage sludge biochars. Differences in toxic metal contents of faecal sludge and sewage sludge may not be critical as metals entrained within the biochar are in immobile and stable forms. Consideration needs to be paid to ensure that high pyrolysis temperatures do not increase toxic metal concentration in biochars to greater than the recommended guidelines for toxic metals in soils.

The low CEC and surface area of faecal sludge biochars reviewed here indicate its potential as a soil amendment in soils with low water retention and low CEC values is limited. However, properties of FS biochar including high pH, high ash content and nutrient concentration indicates its potential to improve soil health and crop yield in acidic, low nutrient soils. Faecal sludge biochar also has potential as a slow-release fertilizer due to significant amount of macro-and micronutrients contained within these biochars that increase with increasing pyrolysis temperature. However, further research into the effect of micronutrients within faecal sludge biochar on soil fertility and plant growth is required.

Evaluating the properties of faecal sludge biochar is difficult due to the different technologies used in collection, storage, and transportation of the feedstock. Also of note are differences in faecal sludge characteristics based on location, climate, age of the sludge, type of sanitation technology and seasonality. These factors contribute to the difficulty in describing faecal sludge biochar properties in general terms, and there is an argument to be made that characteristics of large-scale faecal sludge biochar should be determined on a case-by-case basis.

Future research should concentrate on short-term and long-term field studies of sludge biochar application to acidic soils and the potential effect of micro-nutrients such as silicon on crop stress.

## Data Availability

Data sharing is not applicable to this article as no new data were created or analysed in this study.
